# Supplementary material for: How health literacy relates to venous leg ulcer healing: A scoping review
Source: PLoS One. 2023 Jan 18;18(1):e0279368. doi: 10.1371/journal.pone.0279368 (PMC9847895; doi:10.1371/journal.pone.0279368)
Supplement: S1 Appendix — (DOCX) [file pone.0279368.s002.docx]

**Appendix 1: Search strings for MEDLINE via Ovid**

- - 1. exp Leg Ulcer/
    2. (venous leg ulcer* or Venous ulcer* or VLU or Venous insufficiency or Leg ulcer* or Varicose ulcer*).mp. [mp=title, abstract, original title, name of substance word, subject heading word, floating sub-heading word, keyword heading word, organism supplementary concept word, protocol supplementary concept word, rare disease supplementary concept word, unique identifier, synonyms]
    3. 1 or 2
    4. Program Evaluation/
    5. exp Nursing Research/
    6. health education/ or consumer health information/ or health literacy/ or health promotion/ or healthy people programs/ or patient education as topic/ or teach-back communication/
    7. health facilities/ or hospital units/ or hospitals/ or hospitals, community/ or hospitals, general/ or hospitals, high-volume/ or hospitals, low-volume/ or exp hospitals, private/ or exp hospitals, public/ or exp hospitals, rural/ or exp residential facilities/ or health services/ or exp community health services/ or health services for the aged/ or exp nursing care/ or exp nursing services/ or preventive health services/ or exp rural health services/
    8. (Know* adj2 (health or disease or Illness)).mp. [mp=title, abstract, original title, name of substance word, subject heading word, floating sub-heading word, keyword heading word, organism supplementary concept word, protocol supplementary concept word, rare disease supplementary concept word, unique identifier, synonyms]
    9. (Health adj2 (educat* or literacy or interest or understanding)).mp. [mp=title, abstract, original title, name of substance word, subject heading word, floating sub-heading word, keyword heading word, organism supplementary concept word, protocol supplementary concept word, rare disease supplementary concept word, unique identifier, synonyms]
    10. (Interest* adj2 health interest).mp. [mp=title, abstract, original title, name of substance word, subject heading word, floating sub-heading word, keyword heading word, organism supplementary concept word, protocol supplementary concept word, rare disease supplementary concept word, unique identifier, synonyms]
    11. (Information adj2 (dissemination or access or services or technology)).mp. [mp=title, abstract, original title, name of substance word, subject heading word, floating sub-heading word, keyword heading word, organism supplementary concept word, protocol supplementary concept word, rare disease supplementary concept word, unique identifier, synonyms]
    12. (Teach* adj materials).mp. [mp=title, abstract, original title, name of substance word, subject heading word, floating sub-heading word, keyword heading word, organism supplementary concept word, protocol supplementary concept word, rare disease supplementary concept word, unique identifier, synonyms]
    13. (Health Literacy or Educat* or Health promotion or Nurse-led education).mp. [mp=title, abstract, original title, name of substance word, subject heading word, floating sub-heading word, keyword heading word, organism supplementary concept word, protocol supplementary concept word, rare disease supplementary concept word, unique identifier, synonyms]
    14. Recurrence/
    15. 4 or 5 or 6 or 7 or 8 or 9 or 10 or 11 or 12 or 13
    16. attitude to health/ or health knowledge, attitudes, practice/ or "treatment adherence and compliance"/ or "patient acceptance of health care"/ or patient compliance/ or treatment refusal/
    17. Recurrence/
    18. treatment outcome/ or treatment failure/
    19. Wound Healing/
    20. Secondary Prevention/
    21. Self Care/
    22. Stockings, Compression/
    23. (Adher* adj2 (compression or self care or self management)).mp. [mp=title, abstract, original title, name of substance word, subject heading word, floating sub-heading word, keyword heading word, organism supplementary concept word, protocol supplementary concept word, rare disease supplementary concept word, unique identifier, synonyms]
    24. (Concord* adj2 (compression or self care or self management)).mp. [mp=title, abstract, original title, name of substance word, subject heading word, floating sub-heading word, keyword heading word, organism supplementary concept word, protocol supplementary concept word, rare disease supplementary concept word, unique identifier, synonyms]
    25. (Compl* adj2 (compression or self care or self management)).mp. [mp=title, abstract, original title, name of substance word, subject heading word, floating sub-heading word, keyword heading word, organism supplementary concept word, protocol supplementary concept word, rare disease supplementary concept word, unique identifier, synonyms]
    26. (Adher* or Concord* or Compl* or Impact or Effect or Outcome or Adher* or Healing or Recur* or Compress* or self care or Self manag* or health behavio?r*).mp. [mp=title, abstract, original title, name of substance word, subject heading word, floating sub-heading word, keyword heading word, organism supplementary concept word, protocol supplementary concept word, rare disease supplementary concept word, unique identifier, synonyms]

27 16 or 17 or 18 or 19 or 20 or 21 or 22 or 23 or 24 or 25 or 26

28 3 and 15 and 27
