# Supplementary material for: How health literacy relates to venous leg ulcer healing: A scoping review
Source: PLoS One. 2023 Jan 18;18(1):e0279368. doi: 10.1371/journal.pone.0279368 (PMC9847895; doi:10.1371/journal.pone.0279368)
Supplement: S1 File — (ZIP) [file pone.0279368.s003.zip › Sup file 3_NIHtoolGonzalez 2017.docx]

**Supplementary file 3.** **The National Institutes of Health (NIH) quality assessment tool of controlled intervention study**

**Study title and citation:** The Effect of a Patient Education Intervention on Knowledge and Venous Ulcer Recurrence: Results of a Prospective Intervention and Retrospective Analysis (Gonzalez, 2017)

| **The National Institutes of Health (NIH) quality assessment tool of controlled intervention study**  **Website:** https://www.nhlbi.nih.gov/health-topics/study-quality-assessment-tools | | | |
| --- | --- | --- | --- |
| Major Components | Response options | | |
| 1. Was the study described as randomized, a randomized trial, a randomized clinical trial, or an RCT? | Yes | **No** | Cannot Determine/ Not Applicable/ Not Reported |
| 2. Was the method of randomization adequate (i.e., use of randomly generated assignment)? | Yes | **No** | Cannot Determine/ Not Applicable/ Not Reported |
| 3. Was the treatment allocation concealed (so that assignments could not be predicted)? | Yes | **No** | Cannot Determine/ Not Applicable/ Not Reported |
| 4. Were study participants and providers blinded to treatment group assignment? | Yes | **No** | Cannot Determine/ Not Applicable/ Not Reported |
| 5. Were the people assessing the outcomes blinded to the participants' group assignments? | Yes | **No** | Cannot Determine/ Not Applicable/ Not Reported |
| 6. Were the groups similar at baseline on important characteristics that could affect outcomes (e.g., demographics, risk factors, co-morbid conditions)? | **Yes** | No | Cannot Determine/ Not Applicable/ Not Reported |
| 7. Was the overall drop-out rate from the study at endpoint 20% or lower of the number allocated to treatment? | **Yes** | No | Cannot Determine/ Not Applicable/ Not Reported |
| 8. Was the differential drop-out rate (between treatment groups) at endpoint 15 percentage points or lower? | Yes | No | Cannot Determine/ Not Applicable/ **Not Reported** |
| 9. Was there high adherence to the intervention protocols for each treatment group? | **Yes** | No | Cannot Determine/ Not Applicable/ Not Reported |
| 10. Were other interventions avoided or similar in the groups (e.g., similar background treatments)? | Yes | No | Cannot Determine/ Not Applicable/ **Not Reported** |
| 11. Were outcomes assessed using valid and reliable measures, implemented consistently across all study participants? | **Yes** | No | Cannot Determine/ Not Applicable/ Not Reported |
| 12. Did the authors report that the sample size was sufficiently large to be able to detect a difference in the main outcome between groups with at least 80% power? | Yes | **No** | Cannot Determine/ Not Applicable/ Not Reported |
| 13. Were outcomes reported or subgroups analyzed prespecified (i.e., identified before analyses were conducted)? | Yes | No | **Cannot Determine**/ Not Applicable/ Not Reported |
| 14. Were all randomized participants analyzed in the group to which they were originally assigned, i.e., did they use an intention-to-treat analysis? | Yes | No | Cannot Determine/ **Not Applicable**/ Not Reported |
| **Quality Rating** | Good | Fair | **Poor** |
| Additional Comments (If Poor, please state why):  **This is a non-randomized pre- and post-intervention study.**  **The number of recruited participants is too small to establish the difference between the two groups (n=22; and n=28).**  **The study assessed the participants’ gains in knowledge rather their health literacy scores.** | | | |
