# Supplementary material for: How health literacy relates to venous leg ulcer healing: A scoping review
Source: PLoS One. 2023 Jan 18;18(1):e0279368. doi: 10.1371/journal.pone.0279368 (PMC9847895; doi:10.1371/journal.pone.0279368)
Supplement: S1 File — (ZIP) [file pone.0279368.s003.zip › Sup file 4. NIHtoolFinalyson et al 2010.docx]

**Supplementary file 4. The National Institutes of Health (NIH) quality assessment tool for observational cohort and cross-sectional studies**

**Study title and citation:** The impact of psychosocial factors on adherence to compression therapy to prevent recurrence of venous leg ulcers (Finlayson et al 2019)

| **The National Institutes of Health (NIH) quality assessment tool for observational cohort and cross-sectional studies**  **Website:** https://www.nhlbi.nih.gov/health-topics/study-quality-assessment-tools | | | |
| --- | --- | --- | --- |
| Major Components | Response options | | |
| 1. Was the research question or objective in this paper clearly stated? | **Yes** | No | Cannot Determine/ Not Applicable/ Not Reported |
| 2. Was the study population clearly specified and defined? | **Yes** | No | Cannot Determine/ Not Applicable/ Not Reported |
| 3. Was the participation rate of eligible persons at least 50%? | **Yes** | No | Cannot Determine/ Not Applicable/ Not Reported |
| 4. Were all the subjects selected or recruited from the same or similar populations (including the same time period)? Were inclusion and exclusion criteria for being in the study prespecified and applied uniformly to all participants? | **Yes** | No | Cannot Determine/ Not Applicable/ Not Reported |
| 5. Was a sample size justification, power description, or variance and effect estimates provided? | **Yes** | No | Cannot Determine/ Not Applicable/ Not Reported |
| 6. For the analyses in this paper, were the exposure(s) of interest measured prior to the outcome(s) being measured? | Yes | No | Cannot Determine/ **Not Applicable**/ Not Reported |
| 7. Was the timeframe sufficient so that one could reasonably expect to see an association between exposure and outcome if it existed? | Yes | No | Cannot Determine/ **Not Applicable**/ Not Reported |
| 8. For exposures that can vary in amount or level, did the study examine different levels of the exposure as related to the outcome (e.g., categories of exposure, or exposure measured as continuous variable)? | Yes | No | Cannot Determine/ **Not Applicable**/ Not Reported |
| 9. Were the exposure measures (independent variables) clearly defined, valid, reliable, and implemented consistently across all study participants? | Yes | No | Cannot Determine/ **Not Applicable**/ Not Reported |
| 10. Was the exposure(s) assessed more than once over time? | Yes | No | Cannot Determine/ **Not Applicable**/ Not Reported |
| 11. Were the outcome measures (dependent variables) clearly defined, valid, reliable, and implemented consistently across all study participants? | Yes | No | Cannot Determine/ **Not Applicable**/ Not Reported |
| 12. Were the outcome assessors blinded to the exposure status of participants? | Yes | No | Cannot Determine/ **Not Applicable**/ Not Reported |
| 13. Was loss to follow-up after baseline 20% or less? | Yes | No | Cannot Determine/ **Not Applicable**/ Not Reported |
| 14. Were key potential confounding variables measured and adjusted statistically for their impact on the relationship between exposure(s) and outcome(s)? | Yes | No | Cannot Determine/ **Not Applicable**/ Not Reported |
| Quality Rating | **Good** | Fair | Poor |
| Additional Comments (If Poor, please state why):  **While the quality of this cross-sectional study is good, this study examined the participants’ knowledge and other factors influencing their adherence to compression therapy. Health literacy was not assessed.** | | | |
