# Supplementary material for: How health literacy relates to venous leg ulcer healing: A scoping review
Source: PLoS One. 2023 Jan 18;18(1):e0279368. doi: 10.1371/journal.pone.0279368 (PMC9847895; doi:10.1371/journal.pone.0279368)
Supplement: S1 File — (ZIP) [file pone.0279368.s003.zip › Sup file 5. NIH toolGonzalez 2014.docx]

**Supplementary file 5. The National Institutes of Health (NIH) Quality Assessment Tool for before-after (pre-post) studies with no control group**

**Study title and citation:** Education Project to Improve Venous Stasis Self-Management Knowledge (Gonzalez, 2014)

| Criteria | Yes | No | Cannot Determine  Not Applicable  Not Reported | |
| --- | --- | --- | --- | --- |
| 1. Was the study question or objective clearly stated? | Yes |  | |  |
| 2. Were eligibility/selection criteria for the study population prespecified and clearly described? | Yes |  | |  |
| 3. Were the participants in the study representative of those who would be eligible for the test/service/intervention in the general or clinical population of interest? | Yes |  | |  |
| 4. Were all eligible participants that met the prespecified entry criteria enrolled? | Yes |  | |  |
| 5. Was the sample size sufficiently large to provide confidence in the findings? |  | No | |  |
| 6. Was the test/service/intervention clearly described and delivered consistently across the study population? | Yes |  | |  |
| 7. Were the outcome measures prespecified, clearly defined, valid, reliable, and assessed consistently across all study participants? |  | No | |  |
| 8. Were the people assessing the outcomes blinded to the participants' exposures/interventions? |  |  | | Not applicable |
| 9. Was the loss to follow-up after baseline 20% or less? Were those lost to follow-up accounted for in the analysis? |  |  | | Not reported |
| 10. Did the statistical methods examine changes in outcome measures from before to after the intervention? Were statistical tests done that provided p values for the pre-to-post changes? | Yes |  | |  |
| 11. Were outcome measures of interest taken multiple times before the intervention and multiple times after the intervention (i.e., did they use an interrupted time-series design)? |  | No | |  |
| 12. If the intervention was conducted at a group level (e.g., a whole hospital, a community, etc.) did the statistical analysis take into account the use of individual-level data to determine effects at the group level? |  |  | | Not applicable |
| Quality Rating (Good, Fair, or Poor)  **Poor** | | | | |
| Additional Comments (If POOR, please state why):  **This study was a single (no control) group pre- and post-educational intervention study.**  **The sample size was small – 30 participants.**  **Healing and recurrence outcomes were not measured, but self-reported.**  **The results were not controlled for comorbid conditions that might influence venous leg ulcer healing and recurrence.**  **The participants’ knowledge rather than health literacy was assessed.** | | | | |
