# Supplementary material for: How health literacy relates to venous leg ulcer healing: A scoping review
Source: PLoS One. 2023 Jan 18;18(1):e0279368. doi: 10.1371/journal.pone.0279368 (PMC9847895; doi:10.1371/journal.pone.0279368)
Supplement: Attachment — Submitted filename: revisons.docx [file pone.0279368.s005.docx]

Dear Professor Dimitri Beeckman,

Thank you for your comments and assistance on this manuscript.

Here, we outline our responses to the letter.

Reviewer 2:

*Reviewer #2: All comments have been addressed. However, I suggest that authors consider dividing description of procedures of critical appraisal (add to Methods section) from reporting the quality of evidence (which should stay in Results section).*

Thank you for this comment. We have addressed this by moving those sections into the relevant place, into the methods.

*Typographical errors:*

*line 92, remove one dot (hold. .)*

*line 103, add dot after reference (20).*

*line 106, add dot after reference (17, 20).*

*line 124, add dot after reference (26).*

We have addressed all these by making these exact changes.
